# Graphene Platelets-Based Magnetoactive Materials with Tunable Magnetoelectric and Magnetodielectric Properties

**DOI:** 10.3390/nano10091783

**Published:** 2020-09-09

**Authors:** Ioan Bica, Eugen Mircea Anitas

**Affiliations:** 1 Faculty of Physics, West University of Timisoara, V. Parvan Avenue 4, 300223 Timisoara, Romania; 2 Joint Institute for Nuclear Research, 141980 Dubna, Russia; 3 Horia Hulubei, National Institute of Physics and Nuclear Engineering, 077125 Bucharest-Magurele, Romania

**Keywords:** magnetoactive materials, magnetoelectric effects, magnetodielectric effects, graphene, cotton fabric, silicone oil, carbonyl iron

## Abstract

We fabricate hybrid magnetoactive materials (hMAMs) based on cotton fibers, silicone oil, carbonyl iron and graphene nanoplatelets (nGr) at various mass concentrations $\Phi_{\mathrm{nGr}}$. The obtained materials are used as dielectric materials for manufacturing plane electrical capacitors. The equivalent electrical capacitance $C_{p}$ and resistance $R_{p}$ are measured in an electric field of medium frequency *f*, without and respectively with a magnetic field of magnetic flux density *B* in the range from 0.1 T up to 0.5 T. The results are used to extract the components $\epsilon_{r}^{\prime }$ and $\epsilon_{r}^{\prime \prime }$ of the complex relative permittivity $\epsilon_{r}^{*}$, and to reveal the magnitude of the induced magnetoelectric couplings $k_{x}$ and magnetodielectric effects MDE. It is shown that $\epsilon_{r}^{\prime }$, $\epsilon_{r}^{\prime \prime }$, $k_{x}$ and MDE are significantly influenced by f,B and $\Phi_{\mathrm{nGr}}$. We describe the underlying physical mechanisms in the framework of dipolar approximation and using elements of dielectric theory. The tunable magnetoelectric and magnetodielectric properties of hMAMs are useful for manufacturing electrical devices for electromagnetic shielding of living organisms.

## 1. Introduction

Hybrid composites, in the form of electroconductive textiles and fibers [1,2,3] have attracted in the last years an increased interest, due to their great potential in fabrication of performant medical devices, in conversion and storage of electrical energy, or in electromagnetic shielding of living organisms. An important class of such materials is represented by textiles based on electroconductive metals [4,5,6], since they are highly stable in time and have good electrical and elastic properties, comparable with classical ones.

Common fabrication methods in such materials, thereafter referred to as hybrid electric composites (hECs), include electrophoretic deposition (EPD) [7], DC and pulsed DC-magnetron sputtering (DC and DCP) [8] or electroless silver plating on tetraethoxy silane-bridged fiber glass [9]. However, while EPD, DC and DCP methods require sophisticated equipment, by depositing metals in the form of thin films through electrolysis, uniform and continuous textile coatings can be achieved. As such, silver coated conductive and antibacterial cotton fabric were fabricated in Reference [10], electrical conductive silk fabric also with high antibacterial activity was prepared in Reference [11], while simple and fast fabrication of conductive silver coatings on carbon fabrics has been reported in Reference [12]. The obtained hECs have superior mechanical and electrical properties as compared to textiles based on cotton or silk fibers, and this feature makes them suitable in fabrication of photovoltaic devices.

A second important class of hybrid composites, consists from natural polymeric fabrics (hemp, cotton, bamboo, etc.) or artificial fabrics in which are embedded ferri/ferro-magnetic nano/micro-particles. They are known in the literature as hybrid magnetic composites (hMACs), and are particular useful in manufacturing of high-performance electromagnetic radiation (EMR) absorbers. To this aim, hMACs with enhanced properties have been fabricated in Reference [13], where a fabric coated absorbing material has been obtained on non-uniform fabrics coated absorbing dope containing 85 wt% modified carbonyl iron (CI) powder, that is, CI particles on which are deposited copper particles. The results show that the minimum reflection loss is −8.43 dB in the frequency range of 8–12 Ghz for modified CI powder, while the non-woven fabric absorbing material has a reflection loss of −26 dB at 9.35 Ghz. Also, polyester fabrics based on CI particles and nano carbon black along with aluminium sputtering on polyethylene terephtelate exhibit good microwave absorbing properties, particularly in the range 8.2 ÷ 12.4 Ghz [14], while graphene/Fe nanocomposites have significantly enhanced electromagnetic radiation absorbing properties [15]. In the later case, the maximum reflection loss to electromagnetic waves is up to −31.5 dB at 14.2 Ghz. Other important applications of hMACs can be found in remote controlled drug release [16], treatment of fungal infections and antimicrobial indications [17], enhanced magnetic energy harvesting [18] or for water purification [19].

However, recent technological advancements expose living organisms to unprecedented radiation levels of electromagnetic radiation, sometimes well beyond the limits of many existing hMACs, and therefore, manufacturing of efficient, low-cost and environmental-friendly hMACs became a stringent necessity nowadays. In this work we partially address this issues, and present the fabrication of hMACs, and respectively of several types of hybrid electromagnetic active (hEMACs) materials. The later ones can act both as hECs and hMACs, and are based on cotton fibers, silicone oil, CI and graphene nanoplatelets at different mass concentrations. The obtained hMACs and hEMACs are used as dielectric materials for fabrication of plane electrical capacitors. The corresponding electrical capacitance and resistance are measured in an electric field with frequency between 1 kHz and 1000 kHz, without and with an external magnetic field.

The results show that magnetoelectric and magnetodielectric effects (MDEs) are induced in both hMACs and hEMACs, and they are sensibly influenced by the mass concentration of graphene nanoplatelets, by the frequency of the electric field and magnetic flux density. The physical mechanisms which lead to the observed effects are described in terms of magnetic dipolar approximation and using elements of linear dielectric theory. The obtained results can be useful in fabrication of high performance low- and medium-frequency electromagnetic radiation absorbers and magnetic/electric field sensors.

## 2. Materials and Methods

**Figure 1:**
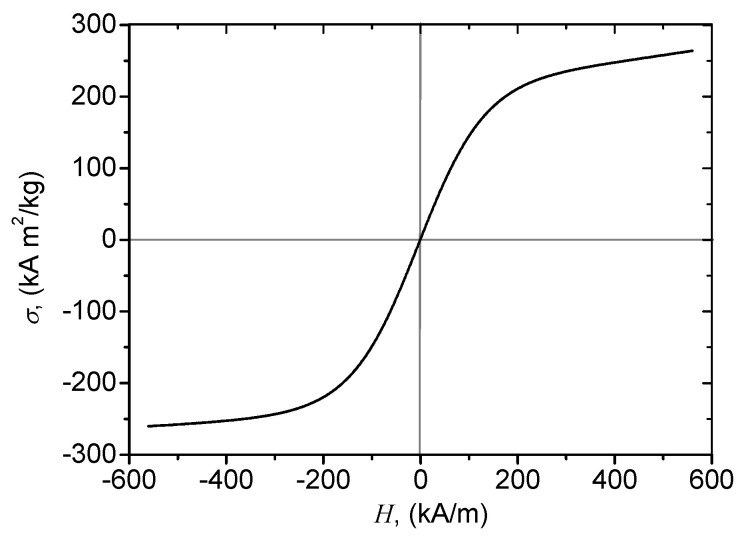
Magnetization curve of carbonyl iron (CI) microparticles. The measurements were performed under sine waveform driving field conditions by means of a laboratory made ac induction hysteresis graph described in Reference [20].

The materials used for fabrication of hMACs and hEMACs are:

- CI microparticles from Sigma-Aldrich (St. Louis, MO, USA) (product number C3518), with an average diameter of 5 μm and density 7.86 g/cm^3^ at 25 °C. The saturation magnetization of the microparticles is 245 Am^2^/kg at intensities H≥0.45 kA/m (Figure 1).
- Graphene nanoplatelets nGr Sigma-Aldrich (product number 900394) with a height of few nm, length about 2 μm, surface area 300 m^2^/g, and densities in the range 0.2 ÷ 0.4 g/cm^3^. The pure graphitic composition makes nGr excellent electrical and thermal conductors and can improve mechanical properties such as stiffness, strength, and surface hardness of the matrix material. In addition, they are compatible with almost all types of natural and synthetic polymers, and can be an active ingredient in inks or coatings as well as an excellent additive to plastics of all kinds.
- Silicone oil (SO) from Siliconi Comerciale SpA (MS100 type) with density 0.97 g/cm^3^, dynamic viscosity $\eta_{0}=0.7$ mPa×s, and relative dielectric permittivity $\epsilon_{r\mathrm{SO}}^{\prime }=2.8$ at 25 °C.
- Gauze bandage (GB) from Medicomp (type 4) based on cotton fibers with granulation of 30 g/cm^2^.

The main steps used in preparation of hMACs and hEMACs are the following:

1. One prepares at 150 °C for 300 s, polyphasic liquids $L_{i}$, i=0,1,2,3 based on CI, nGr and SO with mass weights and concentrations given in Table 1.
2. One cuts four pieces of GB, each one with dimensions 30 mm × 30 mm. Their height is 1.40 mm and the weight of each piece is 0.25 g
3. On each GB is poured 1 g of $L_{i}$, and a hMAC is obtained (for i=0), and hEMACs are obtained for i=1,2,3. The mass fractions of each component in hMAC and hEMACs are indicated in Table 2, and their dimensions coincide with those of GB.

**Figure 2:**
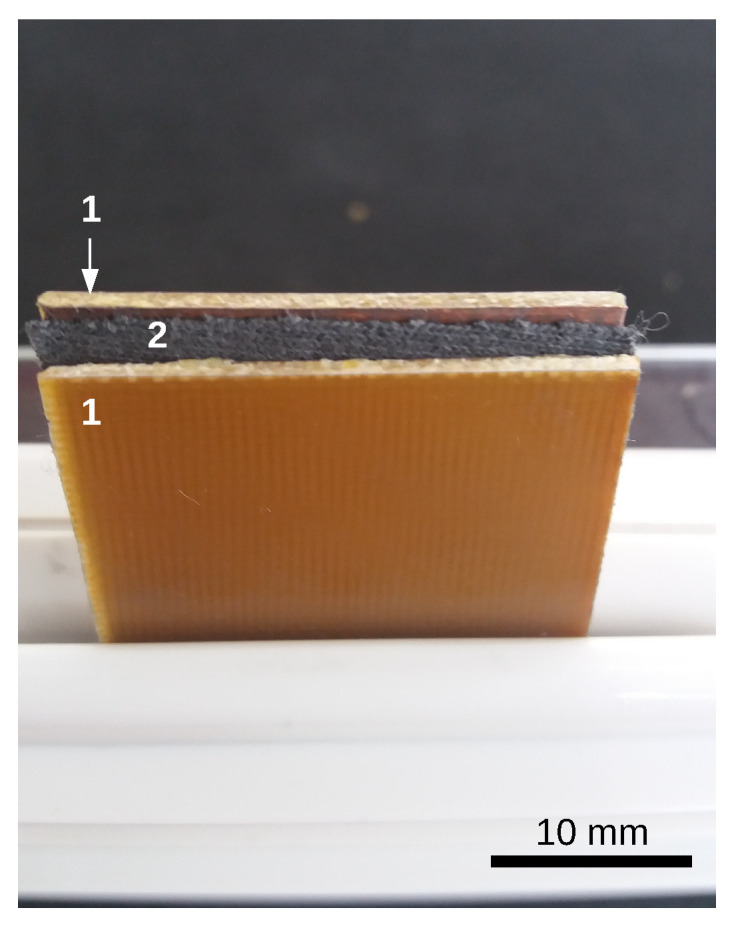
The electrical device ED. 1-textolite plates; 2- hMACs or hEMACs.

The electrical device (ED) is a plane electrical capacitor with hMACs, and respectively hEMACs as dielectric materials, and is manufactured as follows:

1. One cuts 8 textolite plates, covered with copper on one side, with dimensions 30 mm × 30 mm × 0.5 mm.
2. On four of the copper-coated side textolites are placed hMACs, and respectively ${\mathrm{hEMAC}}_{i}$, with i=1,2,3.
3. The remaining four plates are placed on top of the hMACs or hEMACs, with the copper side inward, that is, in contact with the hMAC or hEMACs. At the end of this procedure one obtains ED, with the geometry shown in Figure 2.

**Figure 3:**
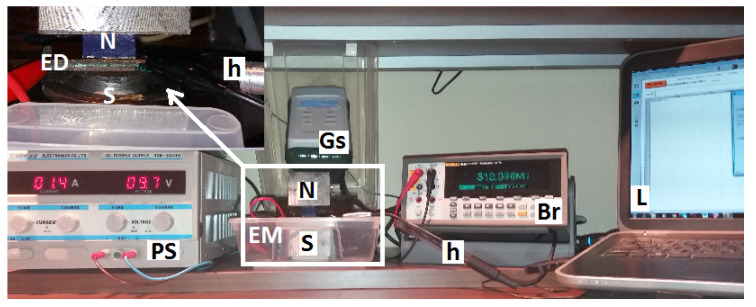
Experimental setup for studying magnetoelectric and magnetodielectric effects (MDEs) in hMACs and hEMACs. N and S-poles of the electromagnet (EM), ED–electrical device, Gs–gaussmeter, h–Hall probe, PS–source of continuous current, Br–RLC bridge, L–computing unit.

The experimental setup used for investigating the MDEs induced in hMAC and hEMACs by the superposition of the magnetic field on the medium frequency electric field, is shown in Figure 3. The setup consists from an electromagnet (EM), the ED and Hall probe h fixed between the N and S poles of the EM, the gaussmeter Gs (DX-102 type), the RLC bridge (E7-20 type), source of continuous current (RXN-3020D type), and the computing unit L with dedicated software for the RS-232C interface.

The hMAC and hEMACs are introduced by turn between the N and S poles of EM. They are electrically connected to the RLC bridge. The voltage at the bridge terminals is 1 Vac, and the impedance is fixed at 100 kΩ. The electrical capacitance $C_{p}$ and resistance $R_{p}$ are measured in the presence of a magnetic field superimposed on a low/medium-frequency electric field. The measurements are recorded at time intervals of 5 s after the magnetic flux density *B* is fixed. This is achieved by changing the electric current intensity provided by the source of continuous current through the coil of EM.

## 3. Results and Discussion

### 3.1. Electrical Capacitance and Resistance

**Figure 4:**
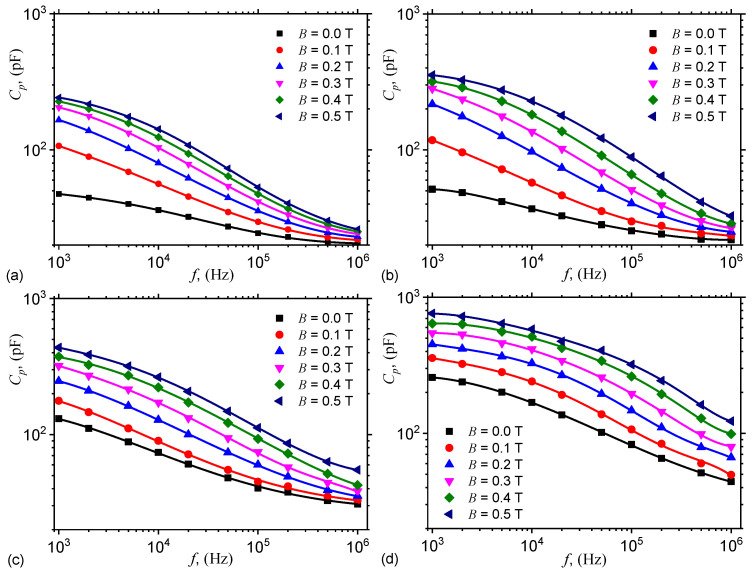
Variation of capacitance $C_{p}$ with frequency *f*, at fixed values of magnetic flux density *B*. (**a**) hMAC. (**b**) hEMAC_1_. (**c**) hEMAC_2_. (**d**) hEMAC_3_.

**Figure 5:**
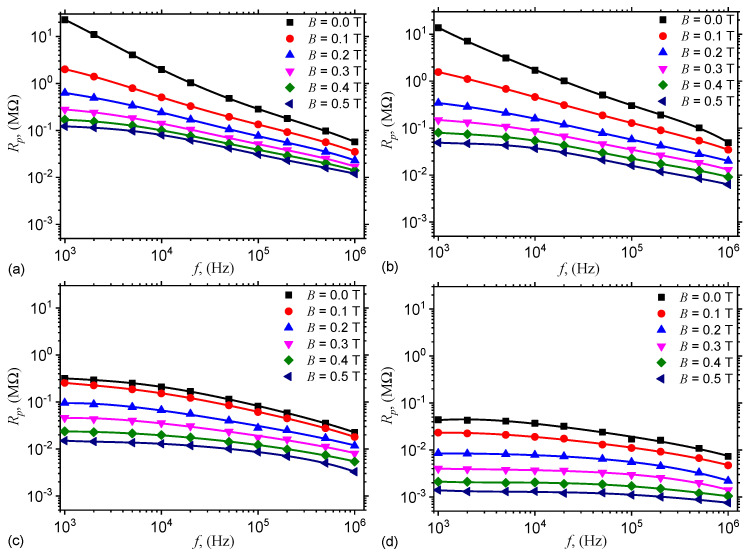
Variation of resistance $R_{p}$ with frequency *f*, at fixed values of magnetic flux density *B*. (**a**) hMAC. (**b**) hEMAC_1_. (**c**) hEMAC_2_. (**d**) hEMAC_3_.

The variation of $C_{p}$ and $R_{p}$ with the frequency *f*, at fixed values of *B* is shown in Figure 4, and respectively in Figure 5. The results show that the variation of capacitance and resistance with frequency *f* at fixed values of magnetic flux density *B*, that is, $C_{p}=C_{p}{\left(f\right)}_{B}$, and respectively $R_{p}\;=\;R_{p}{\left(f\right)}_{B}$ are sensibly influenced by both *f* and *B*, as well as by the mass concentration of graphene nanoplatelets $\Phi_{\mathrm{nGr}}$. In particular, the capacitance increases with $\Phi_{\mathrm{nGr}}$ for fixed values of *f* and *B*. However, for a fixed value of $\Phi_{nGr}$, the capacitance increases with *B*, and decreases with *f*. Similarly, the resistance decreases with $\Phi_{\mathrm{nGr}}$ at fixed values of *B* and *f*.

### 3.2. Theoretical Model

**Figure 6:**
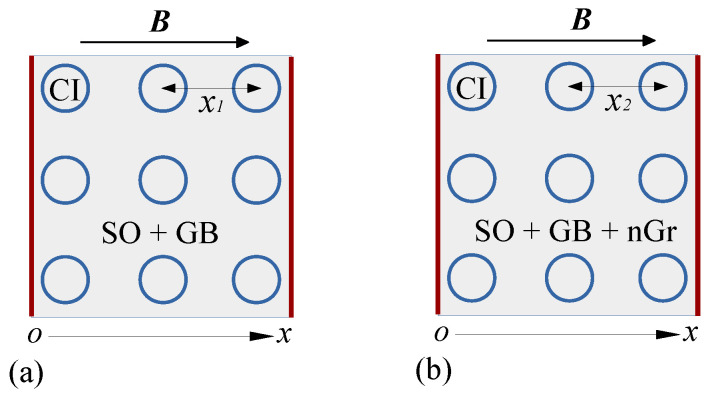
Schematic representation of the arrangement of CI microparticles in hMACs and hEMACs, in the presence of a magnetic field with density *B*. Here, $x_{1}$ and $x_{2}$ are the distances between magnetic dipoles center-of-masses, ox-is the coordinate axis, while SO, GB and nGr are silicone oil, gauze bandage, and respectively graphene nanoplatelets. (**a**) SO + GB (**b**) SO + GB + nGr.

In the presence of a magnetic field, CI microparticles become magnetic dipoles and arrange themselves in chain-like structures oriented along the magnetic field lines. This is schematically illustrated in Figure 6.

Within each chain, dipolar interactions arise between magnetic dipoles. The intensity of such interactions is given by [21,22]:

(1) $$F_{m}=-3\mu_{s}\mu_{0}m^{2}/\left(\pi x^{4}\right),$$

where $\mu_{s}≃1$ is the relative magnetic permeability of SO + GB + nGr, $\mu_{0}$ is the vacuum magnetic constant, *m* is the magnetic dipole moment, and *x* is the distance between the center-of-masses of two neighbouring and identical magnetic dipoles at time t>0, when B≠0. The minus sign in above equation denotes that the dipoles forming the chain are attracted to each other.

At the initial moment, when the magnetic field is applied, the distance δ between the center-of-masses of the dipoles can be written as [21]: $\delta =d/\phi^{-1/3}$, where *d* is their average diameter, and ϕ is the corresponding volume fraction. For the solid phases present inside hMACs or hEMACs, the following relation holds between the volume and mass fractions Φ [23]: $\phi =\left(\rho_{1}/\rho_{s}\right)\Phi$, where $\rho_{1}$ and $\rho_{s}$ are the mass densities of solid, and respectively of the liquid phase from the polyphasic liquid (see Table 1). Thus, for hMAC, at t=0, the distance between the center-of-masses of magnetic dipoles is given by:

(2) $$\delta_{\mathrm{hMAC}}=\frac{d}{{\left(\Phi_{\mathrm{CI}}\frac{\rho_{\mathrm{SO}}}{\rho_{\mathrm{CI}}}\right)}^{1/3}},$$

where $\Phi_{\mathrm{CI}}$ is the mass fraction of CI, and $\rho_{\mathrm{SO}}$ and $\rho_{\mathrm{CI}}$ are the mass densities of SO, and respectively of CI microparticles. A similar relation holds for hEMACs, that is,

(3) $$\delta_{\mathrm{hEMAC}}=\frac{d}{{\left(\Phi_{\mathrm{CI}}\frac{\rho_{\mathrm{SO}}}{\rho_{\mathrm{CI}}}\left(1+\Phi_{\mathrm{nGr}}\frac{\rho_{\mathrm{SO}}}{\rho_{\mathrm{nGr}}}\right)\right)}^{1/3}},$$

where $\Phi_{\mathrm{nGr}}$ is the mass fraction of nGr, and $\rho_{\mathrm{nGr}}$ is the mass density of nGr.

By using numerical values d=5μm, $\rho_{\mathrm{SO}}=0.97$ g/cm^3^, $\rho_{\mathrm{CI}}=7.86$ g/cm^3^, $\Phi_{\mathrm{CI}}=16$ wt%, and $\Phi_{\mathrm{CI}}$ given in Table 2, Equations (2) and (3) give $\delta_{\mathrm{hMAC}}=18.50\;\mu$m, $\delta_{{\mathrm{hEMAC}}_{1}}=18.20\;\mu$m, $\delta_{{\mathrm{hEMAC}}_{2}}=17.91\;\mu$m and $\delta_{{\mathrm{hMAC}}_{3}}=17.64\;\mu$m. This shows that by increasing $\Phi_{\mathrm{nGr}}$, the initial distances between center-of-masses of dipoles decreases with up to 4.65% (for hEMAC_3_), and this has direct consequences on the electrical properties of hEMACs.

#### 3.2.1. Magneto-Electric Couplings

To explain the underlying physical mechanisms, we consider first that the maximum intensity of the dipolar interaction is attained when x=d in Equation (1), that is,:

(4) $$F_{m}^{\max}=-3\mu_{s}\mu_{0}m^{2}/\left(\pi d^{4}\right).$$

However, a resistance force of the type:

(5) $$F_{r}=-k_{s}\left(x-\delta \right),$$

arise from the systems consisting from matrices GB + SO (for hMAC), or GB + SO + nGr (for hEMACs). In the last equation, $k_{s}$ is the magneto-electric coupling between two neighbouring magnetic dipoles and the matrix in which it takes place the interaction, while *x* and δ are the equilibria distances between center-of-masses of two identical and neighbouring magnetic dipoles at t>0, after the magnetic field is applied, and respectively at t=0.

The equilibrium condition between Equations (4) and (5) gives:

(6) $$x=\delta \left(1-\frac{3\mu_{s}\mu_{0}m^{2}}{\pi \delta k_{s}d^{4}}\right),$$

where the magnetic dipolar moment *m* can be expressed as $m=\pi d^{3}B/\left(2\mu_{0}\right)$ [21]. Therefore, the last equation can be rewritten as:

(7) $$x=\delta \left(1-\frac{3\pi d^{2}B^{2}}{4\mu_{0}k_{s}\delta }\right).$$

By using numerical values $\mu_{0}=4\pi 10^{-7}$ H/m, and d=5μm, one obtains:

(8) $$x\left(\mu m\right)=\left\{ \begin{array}{cc} 18.50\left(1-2.533\frac{B^{2}\left(T\right)}{k_{s0}}\right), & \;\mathrm{for}\;\mathrm{hMAC}\\ 18.20\left(1-2.575\frac{B^{2}\left(T\right)}{k_{s1}}\right), & \;\;\;\mathrm{for}\;{\mathrm{hEMAC}}_{1}\\ 17.91\left(1-2.671\frac{B^{2}\left(T\right)}{k_{s2}}\right), & \;\;\;\mathrm{for}\;{\mathrm{hEMAC}}_{2}\\ 17.64\left(1-2.657\frac{B^{2}\left(T\right)}{k_{s3}}\right), & \;\;\;\mathrm{for}\;{\mathrm{hEMAC}}_{3}. \end{array}\right.$$

From an electrical point of view, two neighbouring dipoles from a chain, and which are situated at a distance *x* apart from each other form both an electrical capacitor and an electrical resistor. The capacitance can be assimilated to one of a plane capacitor, and the electrical resistance to one of a linear resistor. Therefore, the capacitance $C_{x}$ and resistance $R_{x}$ of two neighbouring electrical dipoles, can be written as:

(9) $$C_{x}=\pi \epsilon_{0}\epsilon_{r0}^{\prime }d^{2}/\left(4x\right),$$

and respectively

(10) $$R_{x}=4x/\left(\sigma_{0}^{\prime }\pi d^{2}\right).$$

Here, $\epsilon_{0}$ and $\epsilon_{r0}^{\prime }$ are the vacuum dielectric constant, and respectively the relative dielectric permittivity of SO + GB (for hMAC), or of SO + GB + nGr (for hEMACs), and $\sigma_{0}^{\prime }$ is the electrical conductivity of SO + CI (for hMAC) and of SO + CI + nGr (for hEMACs).

Geometrically, hMAC and hEMACs from the ED have the shape of a parallelipiped (see Figure 2) with a thickness h=1.40 mm, and the edges of the common plates surface L=30 mm. Along the height *h*, which coincides with the direction of ox-axis in Figure 6, one considers that the number of dipoles is $n_{d}$. Then, for $n_{d}\gg 1$, the thickness of the capacitor can be approximated by:

(11) $$h=n_{d}x+n_{d}d,$$

and thus, the number of dipoles from a chain can be expressed as:

(12) $$n_{d}=h/\left(d+x\right).$$

By introducing Equation (6) in Equation (12), the number of dipoles inside each chain becomes:

(13) $$n_{d}=\frac{h}{d\left(1+\frac{\delta }{d}\left(1-\frac{3\pi d^{2}B^{2}}{4\mu_{0}k_{x}\delta }\right)\right)}.$$

Thus, by increasing *B*, the empty space along the dipole chains is filled with additional dipoles from within the suspension, and as a consequence, the distance *x* between them decreases. Since the number *n* of dipoles inside the chains can be approximated by $n≃\Phi L^{2}h/V_{d}$, where Φ is the volume fraction of magnetic dipoles, each of volume $V_{d}=\pi d^{3}/6$, one can write:

(14) $$n=\frac{6L^{2}h}{\pi d^{3}}\Phi \frac{\rho_{1}}{\rho_{s}}.$$

Further, by expressing the number of dipole chains as $n_{l}=n/n_{d}$, and using Equations (12) and (14), one can write:

(15) $$n_{l}=\frac{6L^{2}}{\pi d^{2}}\Phi \frac{\rho_{1}}{\rho_{s}}\left(1+\frac{x}{d}\right).$$

This relation shows that $n_{l}$ is influenced by the geometry of ED, the volume fraction of the solid phase, and respectively by the magnetic flux density, since *x* also depends on *B* (see Equation (6)).

Within the model, the electrical capacitance $C_{l}$ of a chain of microcapacitors can be approximated by $C_{l}=C_{x}/n_{d}$. Then, for $n_{d}\gg 1$, and by using the expression of $n_{d}$ from Equation (13), the capacitance becomes:

(16) $$C_{l}=\frac{\pi \epsilon_{0}\epsilon_{r0}^{\prime }d^{2}}{4h}\left(1+\frac{d}{x}\right).$$

Since the capacitance $C_{p}$ of the capacitor can be written as $C_{p}=n_{l}C_{l}$, by using Equations (15) and (16), we obtain:

(17) $$C_{p}=C_{p0}{\left(1-\frac{3\pi d^{2}B^{2}}{4\mu_{0}k_{x}\delta }\right)}^{-1},$$

where $C_{p0}$ is the capacitance of ED when B=0, and is given by $C_{p0}=1.5\epsilon_{0}\epsilon_{r0}^{\prime }\Phi L^{2}\rho_{1}d/\left(h\rho_{s}d\right)$. Similarly, one can obtain an expression of the equivalent electrical resistance of ED, as:

(18) $$R_{p}=R_{p0}\left(1-\frac{3\pi d^{2}B^{2}}{4\mu_{0}k_{x}\delta }\right),$$

where $R_{p0}=h\rho_{s}/\left(1.5\sigma_{0}^{\prime \prime }L^{2}\Phi \rho_{1}d\right)$ is the electrical resistance of ED when B=0.

The magneto-electric coupling $k_{x}$ appearing in Equations (20) and (21) influence the material response when a magnetic and an electric field are applied. $k_{x}$ can be written as a function of the capacitance $C_{p}$, and thus, by using Equation (20) one can write:

(19) $$k_{x}=\frac{3\pi d^{2}B^{2}}{4\mu_{0}\left(1-\frac{C_{p0}}{C_{p}}\right)\delta }.$$

**Figure A1:**
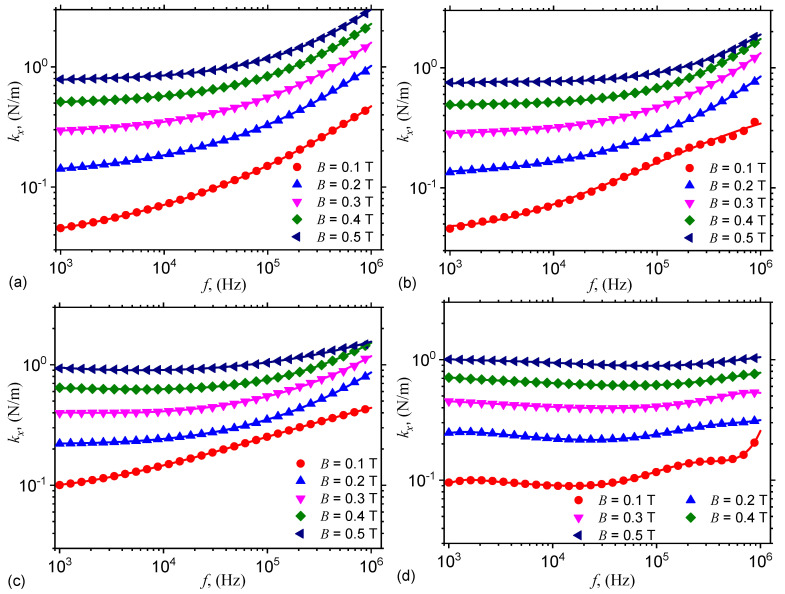
Variation of magneto-electric coupling $k_{x}$ with frequency *f*, at fixed values of magnetic flux density *B*. (**a**) hMAC. (**b**) hEMAC_1_. (**c**) hEMAC_2_. (**d**) hEMAC_3_.

By using numerical values d=5μm, $\mu_{0}=4\pi \times 10^{-7}$ H/m, the values $\delta =\delta_{\mathrm{hMAC}}$ (for hMAC) and $\delta =\delta_{\mathrm{hEMACs}}$ (for hEMACs) obtained above, and the experimental values of the capacitances $C_{p0}$ and $C_{p}$ from Figure 4, one can obtain in Figure A1 the variation of $k_{x}$ with frequency *f*, at fixed values of magnetic flux density *B* (see Figure A1 in Appendix A).

#### 3.2.2. Relative Dielectric Permittivity and Electrical Conductivity

The experimental results in Figure 4 and Figure 5 show that, from an electrical point of view, the capacitor has the structure of an electrical dipole consisting from a plane capacitor connected in parallel with a linear resistor. For such case, the capacitance and resistance can be approximated by:

(20) $$C_{p}=\epsilon_{0}\epsilon_{r}^{\prime }L^{2}/h,$$

and respectively by:

(21) $$R_{p}=h/\left(\sigma^{\prime }L^{2}\right),$$

where $\epsilon_{r}^{\prime }$ is the relative dielectric permittivity.

From the same figures one can see that the capacitors have dielectric losses, and one can write a complex relative dielectric permittivity of the form $\epsilon_{r}^{*}=\epsilon_{r}-j\epsilon_{r}^{\prime \prime }$, where $j=\sqrt{-1}$, and $\epsilon_{r}^{\prime }$ is the dielectric loss factor. By using numerical values $\epsilon_{0}=8.854\times 10^{-12}$ F/m, L=30 mm and h=1.40 mm in Equation (20), one obtains:

(22) $$\epsilon_{r}^{\prime }≃0.176\times C_{p}\left(pF\right).$$

**Figure 7:**
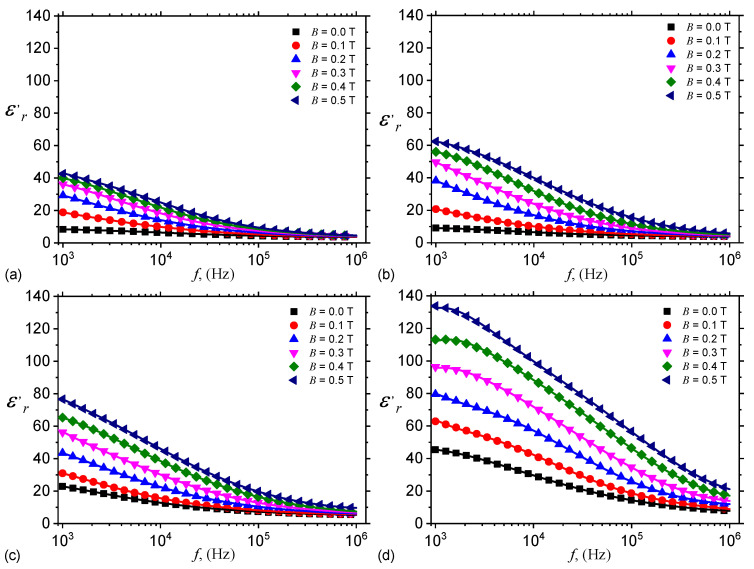
Variation of relative dielectric permittivity $\epsilon_{r}^{\prime }$ with frequency *f*, at fixed values of magnetic flux density *B*. (**a**) hMAC. (**b**) hEMAC_1_. (**c**) hEMAC_2_. (**d**) hEMAC_3_.

Thus, by using the functions $C_{p}=C_{p}{\left(f\right)}_{B,\mathrm{nGr}}$ from Figure 4, one obtains the variation of relative dielectric permittivity $\epsilon_{r}^{\prime }=\epsilon_{r}^{\prime }{\left(f\right)}_{B,\mathrm{nGr}}$ as shown in Figure 7. The results indicate that for the same applied voltage on the ED, the relative dielectric permittivity $\epsilon_{r}^{\prime }$ increases with $\Phi_{\mathrm{nGr}}$, while for a fixed value of $\Phi_{\mathrm{nGr}}$, $\epsilon_{r}^{\prime }$ increases with *B*. The observed effect is due to an increase of the number of charges inside ED. However, by increasing the frequency *f*, $\epsilon_{r}^{\prime }$ decreases at fixed values of *B* and $\Phi_{\mathrm{nGr}}$, and this is due to the changes in the polarization mechanisms which occur in ED. In particular, by increasing *f* up to several kilohertz the dominant polarization is interfacial and of electrical conduction.

Similarly, by using the same numerical values in Equation (21), one obtains the electrical conductivity of hMAC and hEMACs, as:

(23) $$\sigma^{\prime }=\frac{1.555\times 10^{-6}}{R_{p}\left(M\Omega \right)}.$$

**Figure A2:**
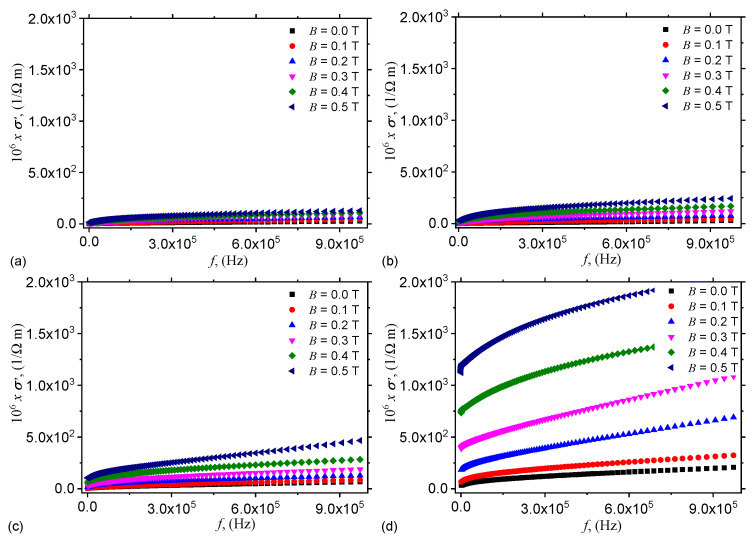
Variation of electrical conductivity $\sigma^{\prime }$ with frequency *f*, at fixed values of magnetic flux density *B*. (**a**) hMAC. (**b**) hEMAC_1_. (**c**) hEMAC_2_. (**d**) hEMAC_3_.

Thus, by using experimental data from Figure 5 in the above equation, one obtains the variation $\sigma^{\prime }=\sigma^{\prime }{\left(f\right)}_{B,\mathrm{nGr}}$. This is shown in Figure A2 in Appendix A.

The charge excess induced by nGr has direct consequences on dispersion characteristics and magnetodielectric effects (MDEs), as we shall see below. To this aim, let us start from the well known relation between the electrical conductivity and dielectric loss factor, that is, $\sigma^{\prime }=2\pi f\epsilon_{0}\epsilon_{r}^{\prime \prime }$. For the same numerical values of $\epsilon_{0},L$ and *h* as those used in obtaining Equation (22), this gives:

(24) $$\epsilon_{r}^{\prime \prime }=\frac{25.165}{f\left(\mathrm{Hz}\right)Rp_{\left(M\Omega \right)}}.$$

**Figure 8:**
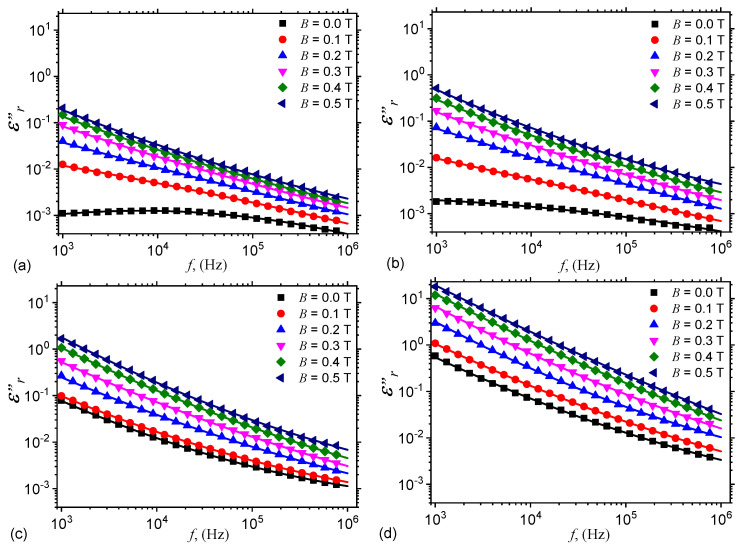
Variation of dielectric loss factor $\epsilon_{r}^{\prime \prime }$ with frequency *f*, at fixed values of magnetic flux density *B*. (**a**) hMAC. (**b**) hEMAC_1_. (**c**) hEMAC_2_. (**d**) hEMAC_3_.

By using the experimental data of $R_{p}$ from Figure 5, one obtains in Figure 8 the variation of $\epsilon_{r}^{\prime \prime }=\epsilon_{r}^{\prime \prime }{\left(f\right)}_{B,\Phi_{\mathrm{nGr}}}$.

The results show that $\epsilon_{r}^{\prime \prime }$ increases with *B* at a fixed value of *f*, due to the increase of the electrical conductivity of hEMACs, as discussed above. However, at fixed *B*, $\epsilon_{r}^{\prime \prime }$ decreases with increasing *f*, due to a decrease of magneto-electric coupling $k_{x}$ (see Figure A1). This can also be seen from Equation (5), where a decrease of the force $F_{r}$ leads to a decrease of phase shift between the polarisation vector of hEMACs and electric field intensity vector.

**Figure A3:**
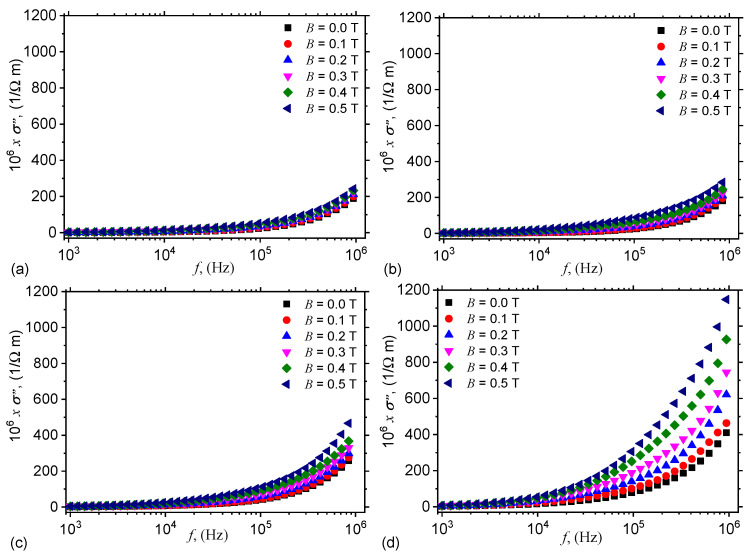
Variation of electrical conductivity $\sigma^{\prime \prime }$ with frequency *f*, at fixed values of magnetic flux density *B*. (**a**) hMAC. (**b**) hEMAC_1_. (**c**) hEMAC_2_. (**d**) hEMAC_3_.

Thus, hMAC and hEMACs can be described by a complex electrical conductivity $\sigma^{*}=\sigma^{\prime \prime }+j\sigma^{\prime \prime }$, where the real part $\sigma^{\prime }$ has been used above to determine $\epsilon_{r}^{\prime \prime }$, arising as a result of the presence of the free pseudocharges [24], while the imaginary part is given by $\sigma^{\prime \prime }=2\pi f\epsilon_{0}\epsilon_{r}^{\prime }$. By using the variation $\epsilon_{r}^{\prime }=\epsilon_{r}^{\prime }{\left(f\right)}_{B,\mathrm{nGr}}$ from Figure 7, one can obtain the variation $\sigma^{\prime \prime }=\sigma^{\prime \prime }{\left(f\right)}_{B,\mathrm{nGr}}$, as shown in Figure A3.

The data for real and imaginary parts of electrical conductivities (Figure A2 and Figure A3) give an absolute value of $\sigma^{*}$ much higher as compared with other systems, such as core-shell structured snowman-like poly (methyl metacrylate) microparticles [25], electrorheological fluids [26], magnetorheological elastomer based on silicone rubber, carbonyl iron and Rochelle salt [27] or in magnetorheological elastomers based on graphene nanoparticles [28].

#### 3.2.3. Magnetodielectric Effects Induced in Hmac and Hemacs

Analysis of the influence of nGr on the dielectric function of the obtained composites is performed by introducing he quantity:

(25) $$MDE\left(\%\right)=\left(\frac{C_{p}^{\mathrm{hEMAC}}{\left(f\right)}_{B,\mathrm{nGr}}}{C_{p}^{\mathrm{hMAC}}{\left(f\right)}_{B,\mathrm{nGr}}}-1\right)\times 100,$$

where $C_{p}^{\mathrm{hEMAC}}{\left(f\right)}_{B,\mathrm{nGr}}$ and $C_{p}^{\mathrm{hMAC}}{\left(f\right)}_{B,\mathrm{nGr}}$ are the electrical capacitances of hEMAC (see Figure 4b–d) and hMAC (see Figure 4a) in the presence of a magnetic field superimposed on a medium-frequency electric field.

**Figure 9:**
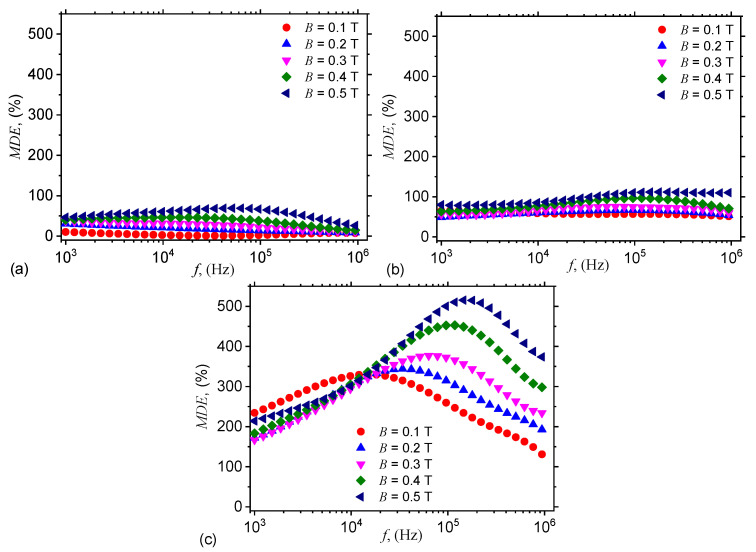
Variation of magnetodielectric effects (MDEs) with frequency *f*, at fixed values of magnetic flux density *B*. (**a**) hEMAC_1_. (**b**) hEMAC_2_. (**c**) hEMAC_3_.

The results are presented in Figure 9, and show that MDEs arise as a combined effects between nGr and CI microparticles from one hand, and the values of magnetic field superimposed on the electric field. The values of MDEs increase with $\Phi_{\mathrm{nGr}}$ and attain a maximum at B=0.5 T for each hEMAC. While for hEMAC_1_ the highest value of MDE is about 70%, and for hMEC_2_ this is about 110%, for hEMAC_3_ the increase is much pronounced, with values about 500%.

According to the proposed model (see Figure 6), the magnetic dipoles are found in the matrix consisting from nGr and SO. By changing the magnetic flux density, the structural ensemble formed by the magnetic dipoles changes also. Moreover, application of a medium-frequency electric field changes also the structure of the GB + SO + CI + nGr leads to changes in the dielectric and electrical functions, as shown in Figure 4 and Figure 5. Addition of nGr induce an increase of charge density, the contribution of dipolar polarisation decreases, and therefore the functions $MDE=MDE{\left(f\right)}_{B,\mathrm{nGr}}$ are characterized by a nonlinear behaviour. Note that the position of maxima and minima in Figure 9 is determined by the frequency *f*.

Similar hMAc and hEMACs with magnetodielectric properties have been obtained din several other works. In particular, in Reference [29] have been obtained hybrid magnetodielectric materials based on magnetorheological elastomers (hMREs) which contain polyurethane sponge foam soaked with magnetorheological suspensions (MRSs) consisting from SO + CI (vol.10%). In the presence of a magnetic field gradient with values up to 1525 kA/m^2^, superimposed on an alternating electric field with frequencies between 20 Hz and 1000 kHz, there have been induced positive MDEs. However, they are about 20% smaller as compared with MDEs observed here (Figure 9). Also in Reference [30] there have been fabricated hybrid MREs based on microfiber cloth soaked with a mixture containing MRSs and silicone rubber (SR. In the same reference it has been shown that the relative dielectric permittivity of the hybrid MRE is strongly dependent on *B*, but with significantly smaller values as compared to those obtained in this work. In Reference [31], hybrid MRSs are prepared by soaking a cotton fabric with a liquid solution based on honey, wax and various concentrations of CI microparticles, and it has been shown that in a static magnetic field with intensities in the range 0.1 ÷ 0.2 T, superimposed on an alternating electric field with frequency of 1 kHz, one obtains positive and negative MDEs. Although the reported values of MDEs are comparable with those obtained in Figure 9, the volume fractions used in Reference [31] are about two times higher as compared to those used here. Good MDEs have been obtained also in MREs based on SR in which are dispersed various types of magnetizable phases. As such, in Reference [32], by using magnetically hard NdFeB fillers as a magnetizable phase, it has been obtained an increase of up to 150% of the MDE as compared to MREs with other types of magnetizable phases, such as Fe or Fe_3_O_4_. However, these values are still significantly smaller than MDEs obtained here for hEMAC_3_.

## 4. Conclusions

In this work we present a low-cost method for fabrication of a new class of magnetoactive materials based on cotton fabrics, SO, CI and nGr at mass concentrations $\Phi_{\mathrm{nGr}}$ from 0 (hMAC), 1.6 wt% (hEMAC_1_), 3.2 wt% (hEMAC_2_) and 4.8 wt% (hEMAC_3_).

It is shown that in the presence of a magnetic field, with magnetic flux density *B*, superimposed on a medium-frequency electric field, the electrical and dielectric functions are sensibly influenced by B,f and $\Phi_{\mathrm{nGr}}$. In particular, the magnetoelectric coupling show an increase of about one order of magnitude, for each concentration $\Phi_{\mathrm{nGr}}$ and in the whole range of frequencies $1\leq f\left(\mathrm{kHz}\right)\leq 10^{3}$, when *B* is increased from 0.1 to 0.5 T. The maximum increase of MDEs occurs when B=0.5 T, and is about 70% for hEMAC_1_ (at f≃60 kHz), 110% for hEMAC_2_ (at f≃100 kHz), and up to 520% for hEMAC_3_ (at f≃150 kHz). A theoretical model, which can describe the physical mechanisms leading to these effects is developed based on magnetic dipolar approximation and using elements of linear dielectric theory.

The obtained results can be useful in fabrication of high performance low- and medium-frequency electromagnetic radiation absorbers and magnetic/electric field sensors.

## Figures and Tables

**Table 1:** The mass *m* (g) and mass fractions Φ (wt%) of CI, silicone oil (SO) and graphene nanoplatelets (nGr) inside the polyphasic liquids $L_{i}$, i=0,1,2,3.

*L_i_*	$m_{\mathrm{CI}}$	$m_{\mathrm{SO}}$	$m_{\mathrm{nGr}}$	$\Phi_{\mathrm{CI}}$	$\Phi_{\mathrm{SO}}$	$\Phi_{\mathrm{nGr}}$
$L_{0}$	1.00	4.00	0.00	20	80	0
$L_{1}$	1.00	3.90	0.10	20	78	2
$L_{2}$	1.00	3.80	0.20	20	76	4
$L_{3}$	1.00	3.70	0.30	20	74	6

**Table 2:** Mass fractions Φ (wt%) of CI, gauze bandage (GB), SO and nGr inside hybrid magnetic composites (hMACs) and hybrid electromagnetic active materials (hEMACs).

Sample	$\Phi_{\mathrm{CI}}$	$\Phi_{\mathrm{GB}}$	$\Phi_{\mathrm{SO}}$	$\Phi_{\mathrm{nGr}}$
hMAC	16	20	64	0
hEMAC_1_	16	20	62.4	1.6
hEMAC_2_	16	20	60.8	3.2
hEMAC_3_	16	20	59.2	4.8

## Appendix A. Magneto-Electric Coupling $k_{x}$ and Electrical Conductivities $\epsilon^{\prime }$ and $\epsilon^{\prime \prime }$

The data in Figure A1 show that $k_{x}$ decreases with increasing $\Phi_{\mathrm{nGr}}$. This is a result of a decrease of mechanical interactions between magnetic dipoles in the SO + GB + nGr matrix, when *B* and *f* are kept fixed.

The results in Figure A2 show that the electrical conductivity increases with *f*, while for a fixed *B* and *f*, it decreases with $\Phi_{\mathrm{nGr}}$. This behaviour can be attributed to the dominance of the dipolar electric polarization over the electronic and interfacial dielectric polarisation when hMAC is in a medium-frequency electric field [24]. In a magnetic field, the dipolar dielectric polarization decreases with increasing *B*, due to the increase of magnetic interactions. Thus, at a given *f*, the electrical conductivity of hMAC decrease with increasing *B*, as indicated in Figure A2a. Addition of nGr leads to the formation of a thin layer of graphene on the surface of CI microparticles. Thus, between the carbon atoms of nGr and the iron atoms forming the surface of CI microparticles, is established a semiconductor-metal contact, which is followed by generation of free electrical pseudocharges inside hEMACs. The net effect is an increase of $\sigma_{r}^{\prime }$ with $\Phi_{\mathrm{nGr}}$ as shown in Figure A2b–d.

The results Figure A3 show that for frequencies f≲2 kHz the imaginary part of $\sigma^{*}$ has a linear variation with *f*, at arbitrarily values of *B* and $\Phi_{\mathrm{nGr}}$. However, in the same frequency range, the real part has a dependence of the type $\sigma^{\prime }\propto f^{n}$ with n>2, and $\sigma^{\prime }\propto B^{2}$. When f≳2 kHz, the imaginary part has also a dependence of type $\sigma^{\prime }\propto f^{n}$ with n>2 and $\sigma^{\prime }\propto B^{2}$. However, now the real part has a variation of the type $\sigma^{\prime }\propto f$, while the variation for which $\sigma^{\prime }\propto B^{2}$ is preserved.
